# G-CSF-Primed Peripheral Blood Stem Cell Haploidentical Transplantation Could Achieve Satisfactory Clinical Outcomes for Acute Leukemia Patients in the First Complete Remission: A Registered Study

**DOI:** 10.3389/fonc.2021.631625

**Published:** 2021-03-15

**Authors:** Yan-Ru Ma, Xiaohui Zhang, Lanping Xu, Yu Wang, Chenhua Yan, Huan Chen, Yuhong Chen, Wei Han, Fengrong Wang, Jingzhi Wang, Kaiyan Liu, Xiaojun Huang, Xiaodong Mo

**Affiliations:** ^1^ Peking University People’s Hospital, Peking University Institute of Hematology, National Clinical Research Center for Hematologic Disease, Research Unit of Key Technique for Diagnosis and Treatments of Hematologic Malignancies, Chinese Academy of Medical Sciences, Beijing Key Laboratory of Hematopoietic Stem Cell Transplantation, Beijing, China; ^2^ Peking-Tsinghua Center for Life Sciences, Beijing, China

**Keywords:** haploidentical donor, acute leukemia, stem cell transplant (SCT), peripheral blood (PB), complete remission (CR)

## Abstract

G-CSF-mobilized peripheral blood (G-PB) harvest is the predominant graft for identical sibling donor and unrelated donor allogeneic hematopoietic stem cell transplantation (HSCT) recipients, but it was controversial in haploidentical related donor (HID) HSCT. In this registry study, we aimed to identify the efficacy of HID G-PB HSCT (HID-PBSCT) for acute leukemia (AL) patients in first complete remission (CR1). Also, we reported the outcomes for the use of G-PB grafts in comparison with the combination of G-BM and G-PB grafts in HID HSCT recipients. Sixty-seven AL patients in CR1 who received HID-PBSCT were recruited at Institute of Hematology, Peking University. Patients who received haploidentical HSCT using the combination of G-BM and G-PB harvests in the same period were enrolled as controls (n=392). The median time from HSCT to neutrophil and platelet engraftment was 12 days (range, 9–19 days) and 12 days (range, 8–171 days), respectively. The 28-day cumulative incidence of neutrophil and platelet engraftment after HSCT was 98.5% and 95.5%, respectively. The cumulative incidences of grade II–IV and grade III–IV acute graft-versus-host disease (GVHD) were 29.9% (95%CI 18.8–40.9%) and 7.5% (95%CI 1.1–13.8%), respectively. The cumulative incidences of total and moderate-severe chronic GVHD were 54.9% (95%CI 40.9–68.8%) and 17.4% (95%CI 6.7–28.0%), respectively. The cumulative incidences of relapse and non-relapse mortality were 13.9% (95%CI 5.4–22.5%) and 3.4% (95%CI 0–8.1%), respectively. The probabilities of overall survival (OS) and leukemia-free survival (LFS) were 84.7% (95%CI 74.7–94.7%) and 82.7% (95%CI 73.3–92.1%) respectively. Compared with the HID HSCT recipients using the combination of G-BM and G-PB grafts, the engraftments of neutrophil and platelet were both significantly faster for the G-PB group, and the other clinical outcomes were all comparable between the groups. In multivariate analysis, graft types did not influence the clinical outcomes. Overall, for the patients with AL CR1, G-PB graft could be considered an acceptable graft for HID HSCT recipients. This study was registered at https://clinicaltrials.gov as NCT03756675.

## Introduction

Allogeneic hematopoietic stem cell transplantation (allo-HSCT) is the most important curative option for patients with acute leukemia (AL). The graft was one of the critical factors for allo-HSCT. Both peripheral blood (PB) and bone marrow (BM) harvests could be used as the graft sources, and cord blood cells could also be used as the graft source. Many studies had compared the clinical outcomes between patients using PB and BM grafts. In patients with human leukocyte antigen identical sibling donors (ISDs), engraftment was faster (1–3), the relapse rate was lower (4), and the leukemia-free survival (LFS) rate was better in the PB group compared with the BM group, particularly for the patients with advanced stage disease (1, 4). Similar results were also observed in patients with HLA-unrelated donors (URDs) (5–7). Considering the fact that PB stem cells (PBSCs) collection is a non-surgical procedure, PBSC transplantation (PBSCT) is more convenient and more acceptable for donors. Thus, PB is the predominant graft source for ISD and URD HSCT. Haploidentical related donors (HIDs) have become the most important alternative donors; however, whether the PB graft is suitable for haploidentical HSCT is controversial. In the HID HSCT regimen using post-transplant cyclophosphamide (PTCY), several prospective studies compared the clinical outcomes between PB grafts and BM grafts. Engraftment was also significantly faster in the PB group; but the difference of the GVHD rates between PB and BM groups was not as significant as those of ISD and URD HSCT recipients. Some studies observed that the LFS rates were significantly poorer in the PB group compared with BM group (8–10); however, the other studies observed that LFS rates of PB group were better than those of BM group (11–13).

Another important HID HSCT regimen was “Beijing protocol”, which proposed by Peking University Institute of Hematology and based on antithymocyte globulin (ATG) (14). “Beijing protocol” had become the most common transplant regimen for HID HSCT in China (15–17). G-CSF primed BM (G-BM) plus G-CSF primed PB (G-PB) harvests were most commonly used in this transplant protocols, but several studies also identified the feasibility of using G-PB harvest alone. Some authors reported that the clinical outcomes of HID HSCT recipients receiving G-PB grafts were satisfactory, however, they were retrospective, single-arm designed studies (18, 19). In a retrospective single-center study, Xu et al. (9) compared the outcomes between patients using G-BM plus G-PB harvests and G-PB alone as grafts in advanced stage [i.e., most of them were beyond the third complete remission (CR3) or in non-remission] AL patients receiving haploidentical HSCT. G-PB group showed no superiority in engraftment compared with G-BM plus G-PB group. In addition, the transplant-related mortality (TRM) was significantly higher and LFS was poorer in G-PB group compared with the G-BM plus G-PB group. In a retrospective multi-center study including all types of hematologic malignancies, Zhao et al. (8) reported that the survival of G-PB groups was poorer than that of the G-BM plus G-PB group. However, this study did not compare the clinical outcomes of G-PB group and G-BM plus G-PB group in AL patients, and the center effect could not be totally excluded either. Thus far, there was no prospective registry study identifying the efficacy of PBSCT in ATG-based HID HSCT. In addition, no prospective study had directly compared the clinical outcomes between G-BM plus G-PB and G-PB alone in AL-CR1 patients receiving HID HSCT. Thus, the role of HID PBSCT in AL-CR1 patients was still unclear.

In the present registry study, we aimed to identify the clinical outcomes of HID PBSCT in AL patients in CR1. We also aimed to compare the clinical outcomes between G-PB alone and G-BM plus G-PB in HID HSCT recipients.

## Patients and Methods

### Study Design

Sixty-seven AL patients in CR1 who received HID PBSCT were recruited in this prospective study at the Peking University People’s Hospital between November 1, 2018, and February 29, 2020. All cases were treated according to the protocol registered at https://clinicaltrials.gov (NCT03756675). The recipients receiving HID HSCT using the combination of G-BM and G-PB harvests (i.e., BM+PB group) in the same period were collected as controls.

**Figure 1:**
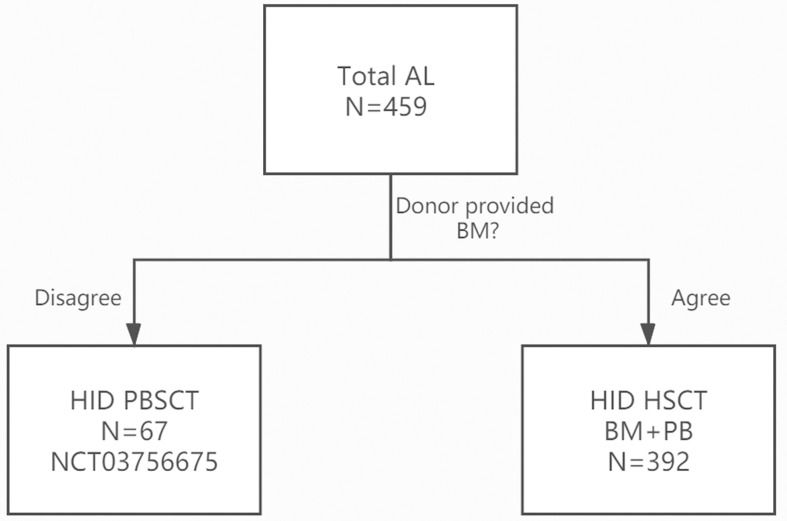
CONSORT (the Consolidated Standards of Reporting Trials) Flow Diagram Showing the Study Design of the trial.

The inclusion criteria: 1) patients aged 2–60 years old; 2) in AL CR1; 3) donors refused the donation of BM; and 4) patients agreed to receive haploidentical PBSCT (**Figure 1**).

The primary endpoint was engraftment rates as defined by neutrophil recovery and platelet recovery. The secondary endpoints include acute graft-versus-host disease (aGVHD), chronic GVHD (cGVHD), relapse, non-relapse mortality (NRM), leukemia-free survival (LFS), and overall survival (OS).

### Transplant Protocols

Conditioning regimens, immunosuppressants, and supportive care have been described in previous studies (20–22). The myeloablative busulfan (BU)-based regimen consisted of (1) cytarabine 4 g/m^2^ for 2 days, busulfan 3.2 mg/kg for 3 days, cyclophosphamide 1.8 g/m^2^ for 2 days, rabbit anti-thymoglobulin 2.5 mg/kg for 4 days, and semustine 250 mg/m^2^ orally for one dose; or (2) cytarabine 4 g/m^2^ for 2 days, busulfan 3.2 mg/kg for 3 days, cyclophosphamide 1.0 g/m^2^ for 2 days, fludarabine 30mg/m^2^ for 5 days, rabbit anti-thymoglobulin 2.5 mg/kg for 4 days, and semustine 250 mg/m^2^ orally for one dose. Five patients received total body irradiation (TBI)-based conditioning. The immunosuppressants included cyclosporine A (CsA), mycophenolate mofetil (MMF), and short-term methotrexate (MTX). G-CSF was administered subcutaneously to patients at 5 ug/kg per day from day +6 until myeloid recovery (23–25).

### Donor Specific Antibodies

Patients were tested for the presence of donor-specific antibodies (DSAs) including class I (i.e., HLA-A, -B, -C) and class II (i.e., HLA-DR) HLA antibodies. Immunoglobulin anti-HLA reactivity in the serum was tested with a bead-based screening assay. Briefly, we used the LABScreen Mixed kit (One Lambda, Canoga Park, CA, USA), which simultaneously detects class I and class II antibodies with microbeads coated with purified class I and class II HLA antigens. For HLA antibody-positive samples with a median fluorescent intensity (MFI) >500, DSAs were further tested using a LABScreen Single Antigen Kit (One Lambda). Above a cut-off value of MFI ≥2000 was considered positive. Patients with positive DSA received rituximab before transplantation, and the co-infusion of umbilical cord blood (26).

### Definitions

The neutrophil engraftment was defined as the first of 3 consecutive days that neutrophils ≥0.5×10^9^/L, and platelet engraftment was defined as the first of 7 consecutive days that platelets ≥20×10^9^/L and transfusion independence. Relapse was defined as BM blasts >5%, or extramedullary manifestation. NRM was defined as death without evidence of leukemia. OS was the period between the date of HSCT and death. LFS was the period between the date of HSCT and relapse or death in remission. GVHD was diagnosed and graded according to internationally accepted criteria (27, 28).

### Statistical Analysis

The last follow-up date was September 1, 2020. Survival was estimated with Kaplan-Meier outcome curves. The cumulative incidences of engraftment, relapse, GVHD were calculated in the completing risk model. The chi-square test, or Fisher’s exact test was used for categorical variables. The non-parametric tests (Mann-Whitney test for two groups, and Kruskal-Wallis tests for more than two groups) were used for continuous variables. The multivariate Cox model was performed to determine the impact of potential prognostic factors on clinical outcomes. Factors included in the regression model were patient age (<30 years *vs*. ≥30 years), gender, donor age (<30 years *vs*. ≥30 years), underlying disease (AML *vs*. others), diagnosis to transplant (≤6 months *vs*. >6 months), HLA mismatching (1 locus vs. ≥2 loci), donor-recipient gender matching (female-male vs. others), ABO compatibility, CD34 count (using median value as a cut-off point), CD3 count (using median value as a cut-off point), and graft source (G-PB vs. G-BM+G-PB). Testing was two-sided at the *P*<0.05 level. Statistical analysis was performed on SPSS software (SPSS, Chicago, IL), and R software (version 2.6.1) (http://www.r-project.org).

## Results

### Clinical Outcomes of HID PBSCT

#### Engraftment

One case had primary graft failure, and her DSA was negative. All the other patients achieved sustained full-donor chimerism. The median time from HSCT to neutrophil engraftment and platelet engraftment was 12 days (range, 9–19 days) and 12 days (range, 8–171 days) after HID PBSCT, respectively. The 28-day cumulative incidence of neutrophil engraftment after HSCT was 98.5% (95%CI 95.1–100%), and the 100-day cumulative incidence of platelet engraftment after HSCT was 95.5% (95%CI 90.1–100%) after HID PBSCT.

#### GVHD

A total of 15 and five patients showed grade II and grade III aGVHD after HID PBSCT, respectively. The 100-day cumulative incidences of grade II–IV and grade III–IV aGVHD after HSCT were 29.9% (95%CI 18.8–40.9%) and 7.5% (95%CI 1.1–13.8%), respectively.

A total of 23, 9, and 2 patients showed mild, moderate, and severe cGVHD after HID PBSCT, respectively. The cumulative incidences of total cGVHD and moderate to severe cGVHD at 1 year after HSCT were 54.9% (95%CI 40.9–68.8%) and 17.4% (95%CI 6.7–28.0%), respectively.

#### Virus Activation

A total of 57 patients showed CMV-DNA after HID PBSCT, and 1 of them developed CMV diseases. The 100-day incidences of CMV-DNA viremia and CMV disease after HID PBSCT were 85.1% (95%CI 76.3–93.8%) and 1.5% (95%CI 0–4.4%), respectively.

A total of five patients showed EBV-DNA viremia, and 2 of them developed posttransplant lymphoproliferative disorders (PTLD) after HID PBSCT. The 100-day cumulative incidences of EBV-DNA and PTLD was 6.0% (95% CI 0.3–11.7%) and 3.0% (95%CI 0–7.1%), respectively.

#### Relapse and NRM

At the last follow-up, 9 patients experienced relapse with a median time of 126 days (range, 53–202 days) after HID PBSCT. The 1-year cumulative incidence of relapse after HID PBSCT was 13.9% (95%CI 5.4–22.5%). In multivariate analysis, female donor/male recipient (FDMR) combination was the only independent prognostic factor for relapse (HR=3.141, 95%CI 1.258–7.840, P=0.014).

At the last follow-up, three patients experienced NRM with a median time of 212 days (range, 36–485 days) after HID PBSCT. The causes of death were summarized in **Supplementary Table 1**. The 1-year cumulative incidence of NRM after HID PBSCT was 3.4% (95%CI 0–8.1%). None of the variables were significantly associated with increased NRM.

#### Survival

The median follow-up among survivals was 341 days (range 177 to 662 days) after HID PBSCT. The probability of OS and LFS at 1 year after HID PBSCT was 84.7% (95%CI 74.7–94.7%) and 82.7% (95%CI 73.3–92.1%), respectively. In multivariate analysis, FDMR combination was the only independent prognostic factor for OS (HR=3.186, 95%CI 1.172–8.660, P=0.023) and LFS (HR=2.911, 95%CI 1.319–6.424, P=0.008).

### Comparison of the Clinical Outcomes Between G-PB Alone and G-BM Plus G-PB in HID HSCT Recipients

#### Patients Characteristics

The characteristics between the patients in the G-PB alone group and G-BM plus G-PB group were summarized in **Table 1** and **Supplementary Table 2**. Most of the variables were comparable between the groups, except that the duration from diagnosis to HSCT was longer in the G-PB groups. As expected, the amounts of mononuclear cells, CD3+ cells, and CD34+ cells in grafts were higher in the G-PB alone groups. DSA testing was positive in 5 (7.5%) patients in the G-PB alone group and 26 (6.6%) patients in the G-PB plus G-BM group.

**Table 1:** Patient characteristics.

Characteristics	G-PB alone	G-PB+G-BM	*P*
	(N = 67)	(N = 392)	
Patient age, years			0.536
Median (range)	30 (2–55)	31 (3–60)	
Sex, *n*(%)			0.644
Male,	42 (62.7)	234 (59.7)	
Female	25 (37.3)	158 (40.3)	
Disease, *n*(%)			0.111
AML	26 (38.8)	200 (51.0)	
ALL	39 (58.2)	185 (47.2)	
MPAL	2 (3.0)	7 (1.8)	
Diagnosis to transplant, months, *n*(%)			0.005
≥6 months	47 (70.1)	202 (51.5)	
<6 months	20 (29.9)	190 (48.5)	
Conditioning regimen, *n*(%)			0.157
BU-based	65 (97.0)	389 (99.2)	
TBI-based	2 (3.0)	3 (0.8)	
Donor age, years			0.236
Median (range)	38 (6–68)	40 (8–65)	
Donor source, *n*(%)			0.631
Father	27 (40.3)	167 (42.6)	
Mother	6 (9.0)	22 (5.6)	
Sibling	14 (20.9)	99 (25.3)	
Child	20 (29.9)	99 (25.3)	
Collateral	0 (0.0)	5 (1.3)	
Donor-recipient ABO match, *n*(%)			0.798
Match	37 (55.2)	215 (54.8)	
Minor mismatch	16 (23.9)	77 (19.6)	
Major mismatch	12 (17.9)	80 (20.4)	
Bidirectional mismatch	2 (3.0)	20 (5.1)	
MNC, ×10^8^/kg			0.001
Median (range)	9.78 (5.52–19.23)	8.91 (3.30–21.31)	
CD34, ×10^6^/kg			0.001
Median (range)	2.70 (1.00–13.52)	2.19 (0.35–9.53)	
CD3, ×10^8^/kg			<0.001
Median (range)	2.72 (1.17–5.25)	1.89 (0.33–7.06)	

AML, acute myeloid leukemia; ALL, acute lymphoblastic leukemia, BM, bone marrow; BU, busulfan; HID, haploidentical donor; MNC, mononuclear cell; MPAL, mixed-phenotype acute leukemia; PB, peripheral blood; TBI, total body irradiation.

#### Clinical Outcomes

**Figure 2:**
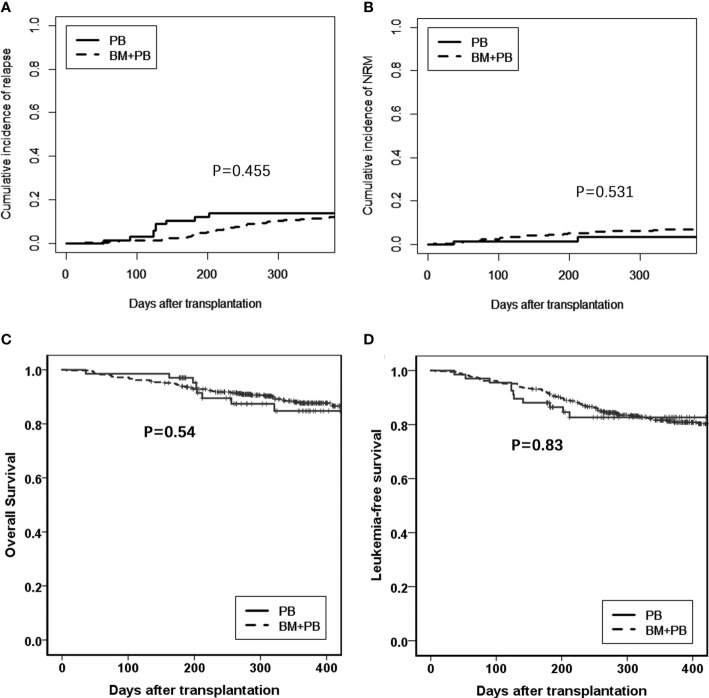
Comparison between G-PB and G-BM plus PB groups **(A)**. Relapse; **(B)**. NRM; **(C)**. Overall survival; and **(D)**. Leukemia-free survival.

The comparison between the G-PB alone group and the G-PB plus G-BM group were shown in **Table 2**. The median time from HSCT to neutrophil engraftment and platelet engraftment was both significantly shorter in the G-PB group compared with the G-BM plus G-PB group [neutrophil: 12 days (range, 9–19 days) versus 13 days (range, 9–25 days), *P*<0.001; platelet: 12 days (range, 8–171 days) versus 15 days (range, 7–268 days), *P*=0.006]. However, all the other outcomes were comparable between the groups (**Figures 2A–D**).

**Table 2:** Cumulative iincidences of clinical outcomes in the G-PB group versus the G-PB plus G-BM group.

	G-PB alone group		G-PB plus G-BM group		*P* ^*^
	Cumulative incidence (%)	95% CI (%)	Cumulative incidence (%)	95% CI(%)	
100-day aGVHD					
Grade II–IV	29.9	18.8–40.9	36.5	31.7–41.2	0.269
Grade III–IV	7.5	1.1–13.8	7.4	4.8–9.9	0.991
1-year cGVHD					
Total	54.9	40.9–68.8	58.3	53.2–63.4	0.794
Moderate to severe	17.4	6.7–28.0	22.4	18.0–26.7	0.571
1-year relapse	13.9	5.4–22.5	11.8	8.5–15.1	0.455
1-year NRM	3.4	0–8.1	6.9	4.3–9.5	0.531
1-year LFS	82.7	73.3–92.1	81.3	77.2–85.4	0.828
1-year OS	84.7	74.7–94.7	87.6	84.1–91.1	0.542

BM, bone marrow; CI, confidence interval; aGVHD, acute graft-versus-host disease; cGVHD, chronic graft-versus-host disease; NRM, non-relapse mortality; LFS, leukemia-free survival; and OS, overall survival; PB, peripheral blood.

*The criterion for statistical significance was P < 0.05.

#### Multivariate Analysis

The results of the multivariate analysis were shown in **Table 3**. Multivariate analyses failed to show significant differences in clinical outcomes between G-PB alone and G-BM plus G-PB groups.

**Table 3:** Multivariate analysis of risk factors for clinical outcomes in total population.

Outcome	HR (95% CI)	P*
**Grade II to IV aGVHD**		
Graft type		
PB plus BM	1	
PB	0.729 (0.452–1.175)	0.195
Other variables		
Donor age		
<30 years	1	
≥30 years	1.511 (1.019–2.242)	0.040
**Grade III to IV aGVHD**		
Graft type		
PB plus BM	1	
PB	0.835 (0.312–2.236)	0.720
Other variables		
Donor age		
<30 years	1	
≥30 years	3.687 (1.296–10.486)	0.014
Donor gender		
Female	1	
Male	2.312 (1.045–5.111)	0.038
CD3 count		
≤2×10^8^/kg	1	
>2×10^8^/kg	2.771 (1.348–5.698)	0.006
**Total cGVHD**		
Graft type		
PB plus BM	1	
PB	0.858 (0.455–1.618)	0.636
Other variables		
HLA mismatching		
1 loci	1	
≥2 loci	2.184 (1.030–4.631)	0.042
**Moderate-severe cGVHD**		
Graft type		
PB plus BM	1	
PB	0.808 (0.425–1.538)	0.517
Other variables		
Patient age		
≥30 years	1	
<30 years	1.534 (1.014–2.319)	0.043
**Treatment failure as defined by overall survival**		
Graft type		
PB plus BM	1	
PB	1.343 (0.655–2.750)	0.421
Other variables		
Donor type		
Others	1	
Female donor to male recipient	2.375 (1.328–4.247)	0.004
**Treatment failure as defined by leukemia-free survival**		
Graft type		
PB plus BM	1	
PB	1.154 (0.622–2.140)	0.649
Other variables		
Donor type		
Others	1	
Female donor to male recipient	1.771 (1.076–2.916)	0.025
**Non-relapse mortality**		
Graft type		
PB plus BM	1	
PB	0.687 (0.205–2.305)	0.543
Other variables		
Donor type		
Others	1	
Female donor to male recipient	2.230 (1.022–4.869)	0.044
**Relapse**		
Graft type		
PB plus BM	1	
PB	1.576 (0.752–3.301)	0.228

aGVHD, acute graft-versus-host disease; BM, bone marrow; CI, confidence interval; cGVHD, chronic graft-versus-host disease; HR, hazard ratio; PB, peripheral blood.

None of variables was significantly associated with increased relapse.

*The criterion for statistical significance was P < 0.05.

## Discussion

This is the first report describing the outcomes of HID PBSCT after the ATG-based conditioning regimen for AL in CR1. This study indicated that hematopoietic recovery for those using G-PB grafts was faster compared with those using G-BM plus G-PB grafts, and GVHD, relapse, NRM, and survivals were similar between groups. This study provided an opportunity for exploring the up-to-date undefined role of HID PBSCT in AL CR1 patients with the ATG-based regimen. To our knowledge, our study represented the first comparison of G-PB alone with G-BM plus G-PB as grafts for HID HSCT in a disease-specific population of patients with AL in CR1.

PBSCT was associated with better engraftment. Randomized studies showed that PB grafts were associated with faster neutrophil and platelet engraftment than BM in ISD and URD HSCT (29, 30). In HID HSCT using post-transplant cyclophosphamide, some studies reported faster engraftment in PBSCT (31, 32). Our analysis also found that neutrophil and platelet engrafted faster in the G-PB group compared with the G-BM plus G-PB group in HID HSCT based on ATG. More rapid hematopoietic recovery of G-PB grafts in HID HSCT may be due to the greater content of mononuclear cells and CD34 cells in PBSC grafts compared with G-BM grafts.

In the present analysis, we did not observe a higher rate of GVHD in the G-PB alone group. As for most studies about ISD and URD HSCT, the rates of cGVHD were reported higher with PB grafts compared to that of BM grafts (1, 33). However, there were also several reports which showed similar rates of cGVHD between PB and BM HSCT (2–4). Our previous study on advanced diseases showed that the G-PB graft was not associated with increased cGVHD when compared with G-BM+G-PB grafts (9). In the present study on AL in CR1, we also observed similar probabilities of cGVHD in G-PB alone and G-BM plus G-PB groups. We speculated that the mature GVHD prophylaxis strategy including ATG in conditioning regimen and long-term schedules of cyclosporin for immunosuppression might reverse the risk of cGVHD with G-PB grafts (34).

Previous observations suggesting cGVHD was associated with graft versus leukemia (GVL) effect in different transplant settings (35, 36), and as mentioned above, more frequent GVHD was observed after PBSCT. Thus, PB grafts may accentuate the GVL effect. Mielcarek et al. (4) observed that among 172 ISD HSCT for hematological malignancies, the 10-year probability of relapse was 20% with PB versus 32% with BM. Bashey et al. (31) analyzed outcomes from a multicenter study comparing HID HSCT with G-CSF-primed PB versus BM and showed the lower relapse risk after PBSCT was limited to patients with leukemia. Several studies also noted that PB grafts had protection against relapse in HID HSCT with PT-CY (7, 11, 31). However, in other studies, PB grafts were not associated with lower rates of relapse (8, 9, 12, 32, 37, 38). Thus, whether more intense GVL effects could be induced in PBSCT remained controversial. In our previous study on advanced diseases, we observed a similar relapse rate between G-PB and G-BM plus G-PB groups (9). One reason may be the comparable incidences of GVHD between G-PB and G-BM plus G-BM groups in the present study, which suggested that G-PB grafts alone could induce a comparable GVL effect with G-PB plus G-BM grafts. On the other hand, because the relapse rate was relatively low among patients with AL in CR1 (20, 39, 40), we could not observe a significantly lower relapse rate in the G-PB group than the G-BM plus G-PB group.

Our previous study showed inferior results after PBSCT on advanced-stage leukemia, as compared to that receiving HID HSCT using G-BM plus G-PB (9). Differences were mostly based on a remarkably higher NRM of 62.5% for PBSCT. This might due to the higher rate of infection and early death in the refractory/relapse diseases. However, the NRM of HID PBSCT was less than 10% in the present study. In addition, the NRM rate of transplants performed in recent years appeared to be lower (mostly less than 20%) than that of transplants done in the previous decade (20, 21). Thus, in these patients with AL-CR1, we did not observe the inferiority of HID PBSCT.

This study was not a randomized designed trial. Thus, it would be premature to derive conclusions regarding the superiority of PBSCT over HID using G-PB plus G-BM in patients with AL in CR1, and these results should be further confirmed by prospective randomized trials.

In summary, this study confirmed the safety and efficacy of HID PBSCT in patients with AL in CR1, and it also suggested that hematopoietic recovery for those using G-PB grafts was faster comparing with those using G-BM plus G-PB grafts, and other clinical outcomes were all comparable between the groups. While BM harvest needed the hospitalization of the donor, trained physicians, and specialized equipment, PBSCs were more convenient and were easy to be collected. For patients with AL in CR1, the G-PB grafts could be used as a reasonable alternative to G-BM plus G-PB grafts in HID HSCT. In the future, these results should be further confirmed by prospective randomized trials.

## Data Availability Statement

The raw data supporting the conclusions of this article will be made available by the authors, without undue reservation.

## Ethics Statement

The studies involving human participants were reviewed and approved by the Institutional Review Board of Peking University. Written informed consent to participate in this study was provided by the participants’ legal guardian/next of kin.

## Author Contributions

XDM and XJH designed the research. Y-RM analyzed the data and wrote the manuscript. All authors provided patient data. All authors contributed to the article and approved the submitted version.

## Funding

This work was supported by National Key Research and Development Program of China (grant number 2017YFA0104500), the Foundation for Innovative Research Groups of the National Natural Science Foundation of China (grant number 81621001), the Key Program of the National Natural Science Foundation of China (grant number 81930004), the Capital’s Funds for Health Improvement and Research (grant number 2018-4-4089), CAMS Innovation Fund for Medical Sciences (CIFMS) (grant number 2019-I2M-5-034), the Science and Technology Project of Guangdong Province of China (grant number 2016B030230003), Peking University Clinical Scientist Program (grant number BMU2019LCKXJ003), and the Fundamental Research Funds for the Central Universities.

## Conflict of Interest

The authors declare that the research was conducted in the absence of any commercial or financial relationships that could be construed as a potential conflict of interest.
