# Supplementary material for: G-CSF-Primed Peripheral Blood Stem Cell Haploidentical Transplantation Could Achieve Satisfactory Clinical Outcomes for Acute Leukemia Patients in the First Complete Remission: A Registered Study
Source: Front Oncol. 2021 Mar 15;11:631625. doi: 10.3389/fonc.2021.631625 (PMC8005750; doi:10.3389/fonc.2021.631625)
Supplement: Supplementary file 1 [file Table_1.docx]

**Supplementary table 1. The Causes of Death**

| **Cause** | **G-PB (N=67)** | **G-BM+G-PB (N=392)** |
| --- | --- | --- |
| **Relapse** | 6 | 21 |
| **Infection** | 1 | 19 |
| **GVHD** | 2 | 6 |
| **TMA** | 0 | 1 |
| **Cerebral hemorrhage** | 0 | 1 |
| **Graft failure** | 0 | 1 |

GVHD, graft-versus-host disease; TMA, thrombotic microangiopathy.
