# Supplementary material for: G-CSF-Primed Peripheral Blood Stem Cell Haploidentical Transplantation Could Achieve Satisfactory Clinical Outcomes for Acute Leukemia Patients in the First Complete Remission: A Registered Study
Source: Front Oncol. 2021 Mar 15;11:631625. doi: 10.3389/fonc.2021.631625 (PMC8005750; doi:10.3389/fonc.2021.631625)
Supplement: Supplementary file 2 [file Table_2.docx]

**Supplementary table 2. Leukemia type**

| **Leukemia type** | **G-PB (N=67)** | **G-BM+G-PB (N=392)** |
| --- | --- | --- |
| **AML** | 26 | 200 |
| **t(8;21)** | 6 | 24 |
| **inv(16)** | 2 | 11 |
| **t(9;11)** | 2 | 6 |
| **t(6;9)** | 0 | 3 |
| **inv(3)/t(3;3)** | 0 | 1 |
| **BCR-ABL1** | 0 | 1 |
| **Mutated NPM1** | 1 | 18 |
| **Biallelic mutation of CEBPA** | 1 | 12 |
| **AML with MDS-related changes** | 1 | 6 |
| **Therapy-related AML** | 1 | 1 |
| **AML, NOS** | 12 | 117 |
| **ALL** | 39 | 185 |
| **B-ALL, t(9;22)** | 9 | 51 |
| **B-ALL, t(v;11q23)** | 0 | 4 |
| **B-ALL, t(1;19)** | 0 | 3 |
| **B-ALL, BCR-ABL1-like** | 0 | 6 |
| **B-ALL, NOS** | 21 | 80 |
| **T-ALL** | 9 | 41 |
| **MPAL** | 2 | 7 |
| **t(9;22)** | 1 | 4 |
| **t(v;11q23)** | 0 | 0 |
| **B/myeloid** | 0 | 0 |
| **T/myeloid** | 0 | 3 |
| **MPAL, NOS** | 1 | 0 |
| **Cycles of induction to achieve CR1** |  |  |
| **1** | 48 | 321 |
| **2** | 15 | 59 |
| **3** | 1 | 8 |
| **4** | 1 | 2 |
| **5** | 1 | 0 |
| **CART*** | 1 | 2 |

ALL, acute lymphoblastic leukemia; AML, acute myeloid leukemia; CART, chimeric antigen receptor T cells; CR, complete remission; MDS, myelodysplastic syndrome; MPAL, mixed-phenotype acute leukemia.

* Three patients who received 2-3 cycles of induction did not reach remission, and achieved complete remission after CART therapy.
